# Gun-Shot Wounds of the Jaw and Facial Injuries

**Published:** 1920-04-10

**Authors:** 


### GUN-SHOT WOUNDS OF THE JAW AND FACIAL INJURIES.

### II. Some Nursing Aspects.

External Treatment.

The care of the external wounds of the face will receive the same surgical attention as other wounds, with such aseptic precaution as may be possible in the circumstances. The condition, like that of the mouth, will be very septic, perhaps gangrenous, and will need the same consistent care by application of compresses of hydrochlorous acid or hot fomentations, as prescribed, until a clean granulating surface is well established. The area of the wounds is often exaggerated in size by reason of the contractile power of the facial tissues, the lacerated edges of which can be traced and gradually brought into correct relationship in view of union or subsequent suturing. At every dressing a careful survey should be given to the location of the small edge flaps, so that in the process of healing their tendency may be toward the correct direction—*i.e.*, the correct contour of the profile and that of the face. As a wound heals its edges thicken and pucker with inward curling, and this should be prevented as much as possible by careful and discreet dissection at every dressing, even by the aid of light massage or smoothing with the tips of the fingers. Various " aids " are devised as supports, which assist in moulding the facial tissues on the correct lines.

Coincident to the nursing work is the specialist treatment; this includes the technical process of fixing the fractured parts into position and reducing displacements by splints devised to prevent and overcome deformities. The rules applying to all fractures apply equally to those of the jaw—complete immobilisation of the broken parts and their correct apposition. In extensive comminution portions of jaw and teeth may be entirely missing; these gaps are bridged and the existing parts on either side are held in place by splints constructed to support the jaw while the wounds are healing. The principles of asepsis are handicapped throughout by the continual growth of the beard and hair of the face, which severely taxes the pride of the surgical nurse. Proficiency in the use of the razor becomes an art when applied to the torn fragments of facial tissue; a skilled barber is a necessary and much valued assistant.

Bandaging.

Knowledge of the correct adjustment of the head bandage to the fractured jaw is of great importance. Its application is a procedure which if incorrectly, carelessly, or negligently applied, and used merely to hold dressings in place, will result in lasting ill-effects, especially in the early stages of the injury. Unless a splint has previously been inserted, the bandage gives no support to the broken parts. Without the support of splints a bandage wrongly applied will, if left in place for a period of time, eventually distort the contour of the lower face from its normal lines by drawing the chin backwards or sideways, and will thus produce a displacement that may become a permanent distortion. Incorrect bandaging will interfere with the circulation, and so deprive the soft tissues of the face of their nourishment, giving rise to further suppuration and sloughing and the formation of adhesions between the mucous surfaces of the lips, the cheeks, and soft tissue over the jaws. The rules of bandaging must therefore be conscientiously observed. In the early stages of the injury it may be necessary to bandage loosely, allowing for free outward drainage and the prevention of unnecessary pain by pressure on the inflamed and sensitive parts, but later bandaging may be firm enough to aid in the support and immobilisation of the jaws. Several types of bandage are in use, such as the four-tailed, or that which is made of webbing 1 inch wide to form a head-gear, having a strap passing under the chin and buckled at one side below the ear. Probably that most generally adopted is the " Barton " bandage, which combines some useful modifications with comfort and firmness, and is to be further recommended for its neatness and security when applied to the most restless patient. It is adjusted as follows : Standing at the right side of the patient, begin at the right occipital and carry upwards and forward over the top of the head down in front of the left ear, passing under the chin up over the head, carry behind to the back of the head. Continue horizontally around the neck forward *across* the chin under the left ear back to the starting-point. When required to hold dressings in place, the horizontal turn may be utilised to cross the upper lip, the nose, or eyes.

Complications.

The results directly following upon a severe gunshot injury to the jaw are usually of a serious nature. All the familiar symptoms of cerebral disturbance may accompany an extensive fracture of the upper jaw involving the higher nerve centres, while the projectile frequently makes a passage through vital structures having an elaborate network of blood-vessels, as, for instance, the neck or under the tongue. Every sufferer from such an injury is liable to secondary hæmorrhage and also to septic infection of the air passages. Sudden hæmorrhage is the most dreaded of complications, and is to be anticipated and provided for in every case. A slight flow of blood, whether coming from the external wounds or within the mouth, should be carefully examined and traced to its source, and the patient kept under strict observation. The adoption of prompt precautionary measures may be the means of averting an otherwise tragic catastrophe. The causes of hæmorrhage are direct injury to the vessels, splinters of bones, and the presence of foreign bodies, with the subsequent spread of sepsis and sloughing of the parts. The location of the bleeding point is most difficult when occurring far back within the mouth, and pressure is made ineffectual by the laceration and œdema of the parts surrounding the artery involved. The patient, unable to open wide the mouth and provide a clear view or space for manipulation, is meanwhile choking and full of panic.

Every accessory known for the use and control of hæmorrhage should, without exception, be near at hand in immediate readiness; the electric head lamp and mirror, several pairs of lingual clamps, hot lotion, adrenalin packing, morphia, and oxygen. Delay means death. Quick action and resource, calm assurance on the part of those in attendance, are as necessary as the remedial agents in securing the quietude of the patient for whom more effectual relief can be accomplished. When all means fail

The previous article appeared on April 3, p. 23.

**Gunshot Wounds of the Jaw**—(*continued*).

to arrest the bleeding it then becomes necessary to tie the carotid artery. After a hæmorrhage, whether slight or severe, skilled after-nursing is most essential. Apart from the condition of shock and collapse, there still remains the primary injury requiring surgical cleanliness; and over the delicate surfaces the vehicle of nourishment has to be conveyed, since the bleeding, if controlled by temporary and not radical means, is very liable to a recurrence. Removal of the foreign bodies and fixation of the fragments is hastened whenever it is possible by the insertion of splints, preserving the immobility of the parts and thus ensuring against a possible recurrence of hæmorrhage.

SEPTIC INVASION OF THE AIR PASSAGES.

Equally common is the incidence of lung infection after an injury to the jaw by micro-organisms invading the bronchi; fully one-fifth of such patients suffer from "septic pneumonia." The predisposing causes are a lowered resistance to disease by shock and previous exposure; the absorption of septic material by suppuration into and from the oral cavity; the inability of the patient by the normal mechanical action to rid the tubes of the accumulation of this septic material and the inspired air passing through infected air passages (nearly all such patients are mouth-breathers). From the time of admission the patient should be examined for chest signs; the temperature, pulse, and respiration recorded at least twice daily. With the appearance of such signs the patient should immediately embark upon the lines of treatment as for pneumonia and be placed in an equable atmosphere, bed screened, and well propped up with pillows. He must be assisted to clear the tubes of the mucus and pus rising into the mouth, which, owing to his disability, he cannot do for himself. A plentiful supply of cotton-wool pledgets on a pair of forceps should be near at hand, and the mouth irrigated as frequently as possible. If he proceeds through the varying stages of pneumonia he is a very sick man, for his malady is liable to the onslaught of gangrene of the lung. It will be fully appreciated that in conjunction with his disability affecting the respiratory tract at its origin, and also probably deprived of speech, his condition calls for qualified, skilful, unremitting care of expert nursing.

Bronchitis, both chronic and acute, is a frequent accompaniment; and also pleurisy, usually due to the presence of fractured ribs. Another factor largely responsible for the chest condition is the administration of anæsthetics or narcotics. The latter given for the relief of pain or delirium greatly predispose to the absorption of septic material without the accompanying elimination of poisons from the body while under its influence; and also when anæsthetised for operation upon the septic mouth, the patients are especially liable to lung complications. Together with the generally septic condition, abscesses and necrosis occur, and can in a large measure be controlled by a strict hygiene of the mouth until all suppuration has ceased and the decomposing sloughs and fragments have come away.

Mild forms of gastritis and disturbed digestion are to be numbered among the ills of a "jaw" patient owing to the inactivity or destruction of the salivary glands; the food passes directly into the stomach unmasticated and unsalivated.

